# Machine Learning Assisted Classification of Aluminum Nitride Thin Film Stress via In-Situ Optical Emission Spectroscopy Data

**DOI:** 10.3390/ma14164445

**Published:** 2021-08-08

**Authors:** Yu-Pu Yang, Te-Yun Lu, Hsiao-Han Lo, Wei-Lun Chen, Peter J. Wang, Walter Lai, Yiin-Kuen Fuh, Tomi T. Li

**Affiliations:** 1 Department of Mechanical Engineering, National Central University, Taoyuan 32001, Taiwan; yupu.yang@g.ncu.edu.tw (Y.-P.Y.); deyun57@gmail.com (T.-Y.L.); a108323045@g.ncu.edu.tw (H.-H.L.); j1997318@gmail.com (W.-L.C.); tomili@ncu.edu.tw (T.T.L.); 2 Delta Electronics Inc., Taoyuan 32063, Taiwan; PETER.J.WANG@deltaww.com (P.J.W.); walter.lai@deltaww.com (W.L.)

**Keywords:** machine learning, aluminum nitride (AlN), principal component analysis (PCA), artificial neural networks (ANNs), in-situ, thin film stress

## Abstract

In this study, we submit a complex set of in-situ data collected by optical emission spectroscopy (OES) during the process of aluminum nitride (AlN) thin film. Changing the sputtering power and nitrogen(N_2_) flow rate, AlN film was deposited on Si substrate using a superior sputtering with a pulsed direct current (DC) method. The correlation between OES data and deposited film residual stress (tensile vs. compressive) associated with crystalline status by X-ray diffraction spectroscopy (XRD), scanning electron microscope (SEM), and transmission electron microscope (TEM) measurements were investigated and established throughout the machine learning exercise. An important answer to know is whether the stress of the processing film is compressive or tensile. To answer this question, we can access as many optical spectra data as we need, record the data to generate a library, and exploit principal component analysis (PCA) to reduce complexity from complex data. After preprocessing through PCA, we demonstrated that we could apply standard artificial neural networks (ANNs), and we could obtain a machine learning classification method to distinguish the stress types of the AlN thin films obtained by analyzing XRD results and correlating with TEM microstructures. Combining PCA with ANNs, an accurate method for in-situ stress prediction and classification was created to solve the semiconductor process problems related to film property on deposited films more efficiently. Therefore, methods for machine learning-assisted classification can be further extended and applied to other semiconductors or related research of interest in the future.

## 1. Introduction

Aluminum nitride (AlN) is a typical semiconductor material that belongs to group III-V with a hexagonal, close-packed wurtzite structure [1]. AlN is a material with high mechanical, thermal, and chemical stability. From an electrical point of view, AlN is well known for its large energy bandgap, high thermal conductivity, high breakdown energy, high resistivity, and high surface acoustic wave (SAW) velocity [2]. AlN has some clearly labeled crystal orientations, such as (100), (101), and (002). The latter parameter is the highest orientation in the (002) plane, prompting research interest in the piezoelectric area [3]. Various methods can produce thin films but sputtering, with the low-cost and low-temperature requirements, is fast becoming the most suitable fabrication method [4]. During the DC sputtering, the arcing phenomena can lead to target poisoning and damage to the target as well as affect thin film deposition afterwards. Differently, RF sputtering will not cause target poisoning, but it has lower deposition rates and imposes a greater cost in terms of RF power supply. Therefore, pulsed DC sputtering is regarded as a deposition method combined with DC sputtering and RF sputtering advantages [5,6].

In the semiconductor industry, optical emission spectroscopy (OES) has become one of the most frequently used in-situ process monitoring tools with the advantage of non-invasiveness. Nevertheless, OES provides much information, unlike other spectra, becoming a challenge for data analysis [7]. During AlN sputtering, the emission peaks of OES mainly consists of Al (300–400 nm), Al^+^ and Ar^+^ (450–600 nm), N (650–700 nm), and Ar (700–850 nm), reflecting the complexity of plasma statues in the chamber [8]. After deposition, ultrahigh-resolution cold field emission scanning electron microscope (CFE-SEM) can be used to characterize deposited thin film [9,10], X-ray diffraction spectroscopy (XRD) can be used for a survey of crystallization [11], and high-resolution transmission electron microscope (HR-TEM) can be applied for the confirmation of microstructure and crystallization [12]. Additionally, thin film residual stress can be measured by XRD results [13,14]. In a recent review, thin film stress was discussed. Thin film stress can be affected by many complex factors like microstructures and deposition parameters. It is noted that the film stress is an important characteristic associated with film strain engineering, which will affect the semiconductor device performance and needs to be mitigated and readjusted in relation to process parameters for increasing the electron mobility in devices [15]. Due to the change of critical power, residual stress from tension to compression can be observed [16]. In the process of film growth, large residual stress will be generated, which will significantly affect the performance of the film. Some papers have studied the relationship between film residual stress and microstructure [17].

In recent years, machine learning for data analysis has become popular in many areas. It can calculate and analyze big data quickly and effectively through the computer system. In biomedicine, some scholars have tried to use Raman spectroscopy to detect nasopharyngeal carcinoma with principal component analysis (PCA) [18]. Some researchers have applied PCA on shear strength perdition [19]. In the area of energy, some researchers use multi-layer perceptron (MLP) to study infrared radiation temperature and concentration [20]. In materials, artificial neural networks (ANNs) have been used to classify graphitic surface exposure to defined environments at different times [21]. In the semiconductor area, in-situ prediction is utilized for Czochralski silicon growth using machine learning [22].

PCA is a multivariate machine learning analysis method based on feature vectors, using an orthogonal transformation to change the observation direction of correlated variables to unrelated variables [23]. Multilayer perceptron (MLP) with multiple hidden layers is a kind of feed-forward deep neural network (DNN) with the most straightforward architectures and most direct input, and the depth is dependent on the stacking of hidden layers [24]. In the machine learning area, compared to the support vector machine (SVM) and radial basis function neural network (RFBNN), two similar methods for classification, MLP has demonstrated better performance in many cases [25].

This study aims to establish a novel method for film residual stress prediction and classification with in-situ OES data. Machine learning approaches can accurately predict the character of deposited film stress based on in-situ optical spectra taken during the sputtering process, and it is more efficient to use MLP models that first filter the data through PCA.

## 2. Materials and Methods

### 2.1. Thin Films Deposition

**Figure 1:**
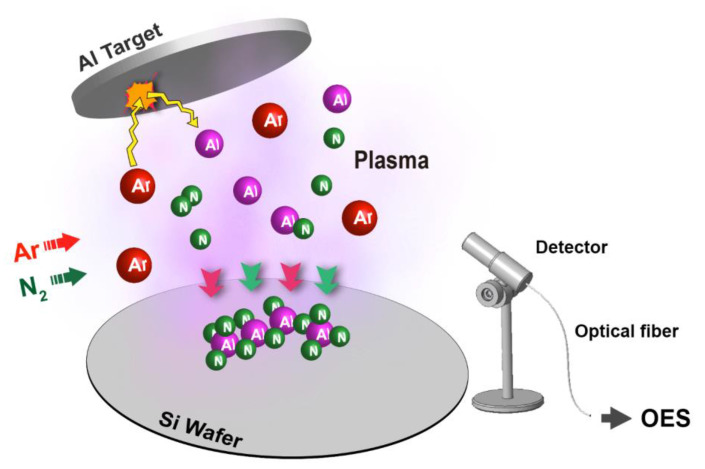
Plasma process reaction during reactive deposition in a sputter chamber.

In this study, experimentally, AlN thin film was deposited on Si (100) p-type substrates using a pulsed DC reactive sputtering with a power supply (HPP-20KA01KAT, Delta Electronics, Taoyuan, Taiwan), as shown in Figure 1, and the Al target we use is 4 inches in diameter with 6 mm thickness in 99.999 wt% purity. During the processes, the mixture gas of nitrogen (N_2_) and argon (Ar) in a ratio from 1:1 to 4:1 was used. The sputtering power of the process is from 400 W to 1000 W, and the pulse frequency was 250 kHz with a 0.6 μs reverse time. Samples were cut into squares with a side length of 2 cm and cleaned in the order of acetone (ACE), isopropanol (IPA), and de-ionized (DI) water. Before the sample was loaded into the chamber, diluted 1.2% hydrofluoric acid was used to remove the oxide from the samples.

### 2.2. Data Acquirement

**Figure 2:**
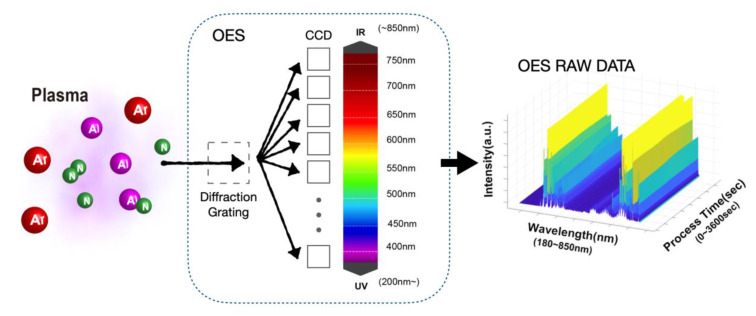
Methodology of OES data acquirement and a waterfall plot showing raw data continuously collected by the spectrometer during a 60-min process.

During the 60-min processes, under the electric field, N_2_ dissociates, and Ar impacts the target to sputter Al from the target. Atoms are partially ionized and emit light. The plasma consists of some radicals, e.g., Al^+^, Al^0^, Ar^+^, Ar^0^, N^+^, and N^0^, and spectra were collected by OES (SE2020-025-FUVN, OtO Photonics, Taoyuan, Taiwan) presented by the emission line, but N^+^ is not detected due to low intensity [8]. A waterfall plot was plotted to show the raw data of one experiment in Figure 2. OES supports the spectral analysis of a 2048-pixel charge-coupled device (CCD). The emission light is transmitted through a diffraction grating and then collected by CCD. The OES device has a spectral decomposition capability of wavelength between 180 and 850 nm, and the resolution between these wavelengths is 1900 pixels.

On the right side of Figure 2, every data “point” reflects an arbitrary unit (a.u.) density in a specific time and wavelength. Spectral intensity is recorded numerically on these pixels, and we call each intensity value a “point”. We use OES to continuously collect the plasma emission spectrum at the interval of 3 s, and the integration time of the spectrum is 25 ms. An integration time (25 ms) can be easily understood as the exposure time of a camera. Over 25 ms of integration time, an OES spectrum is obtained. We call a spectrum at a time point as a “set”. These sets reflect the spectrum at a particular time and are fed into the PCA one by one as input data. After a 60-min experiment, we will receive 1200 sets, which we will call a “group”. Before using this set of data, we used PCA to denoise this set of data.

### 2.3. Flim Charaterization and Stress Calculation

After films being deposited, XRD (D8 Advance, Bruker, Billerica, MA, USA) was used to measure the film texture, CFE-SEM (SU8200, HITACHI, Tokyo, Japan) was used to take the cross-section images of the thin film and the interface between AlN and Si, and HR-TEM (JEM2100, JEOL Ltd., Tokyo, Japan) was used to take the bright-field images and selected area electron diffraction (SAED) pattern. We calculate the residual stress on the thin film using the XRD measurement results and the following using the side-inclination method [13,14]:

(1) $$\sigma =-\frac{1}{2}\cdot \cot\left(\theta_{0}\right)\cdot \frac{E}{1+\upsilon }\cdot \frac{\pi }{180}\cdot \frac{∆2\theta }{∆sin^{2}\psi }$$

where Young’s modulus *E* = 308 GPa and Poisson’s ratio *υ* = 0.287 for AlN [26,27], *σ* is stress, *θ* is diffraction angle, *θ*_0_ is the diffraction angle without stress, and *ψ* is the inclination angle of the sample. After changing the inclination angle of the platform several times, we can obtain the slope curve and an estimated value of the thin film stress [28].

### 2.4. Data Processing and Machine Learning

**Figure 3:**
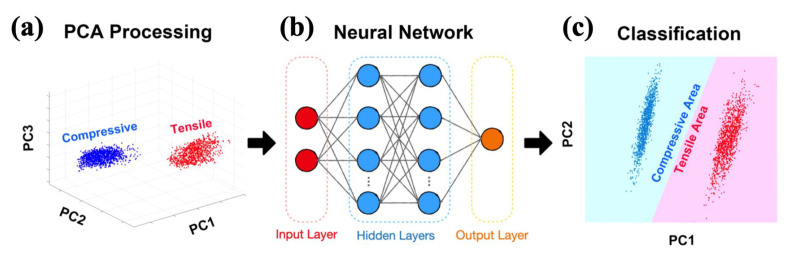
(**a**) After calculating film stress from XRD, we used PCA to help group the data according to compressive and tensile stress, and here shows a subset of the data plotted with respect to the first three principal components from PCA; (**b**) Diagram of MLP—a feed-forward artificial neural network; (**c**) Model based on the neural network to complete the classification.

We combined the different experimental data and analyzed them using PCA, then classified them by calculating stress. In machine learning exercises, PCA is classified as dimension reduction to extract features. In principle, the data dimension is reduced, but the overall performance of the data remains good or not much different. As shown in Figure 3a, PCA can distinguish data through different PCs (principal components). The directions with the largest and the second-largest data differences are extracted from PCA, respectively, called principal component 1 (PC1) and principal component 2 (PC2). Then, we select PC1 and PC2 and input the processed data into MLP. MLP includes at least three nodes (input layer, hidden layers, and output layers), as shown in Figure 3b. The hidden layers are performing the feature extraction of input layer data, which can reduce as well as increase dimension. This process is not designed by the rule of thumb but learned by data. The final output layer is the classified stress (tensile or compressive) [29]. In the end, we have received a machine-determined taxonomy for PC1 and PC2, as shown in Figure 3c. This kind of classification is not necessarily visible, only visible in limited dimensions or selected dimensions. However, the data can be classified to make predictions using this model, whether it is visible or not.

**Figure 4:**
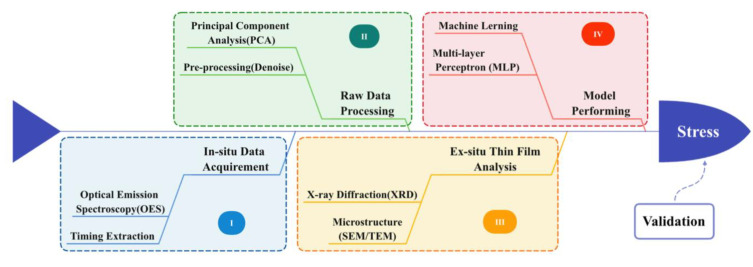
Schematic diagram including four steps in this study.

The above study methods are illustrated as a fishbone diagram, as shown in Figure 4. Through data acquirement, data processing, and thin film analysis (step I-III), a machine learning method is built for stress classification (step IV). Validation experiments after model training can verify if the model can be widely used. The correlation between process parameters and OES has been addressed by varying some of the experimental parameters, which will result in changes in the whole spectrum of OES data that are difficult to quantify or describe. The prediction combined with machine learning is also an innovation of this study which solves the complex problems of plasma composition and energy associated with film deposition and film property.

## 3. Results and Discussion

### 3.1. Stress and X-ray Diffraction Analysis

**Figure 5:**
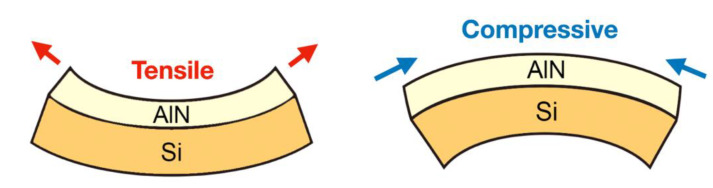
Diagram showing tensile and compressive force acting on the sample during the process.

As shown in Figure 5, there are two kinds of stress acting in the film. In a recent review [15], thin film stress was discussed. Reducing the stress of thin films can improve the electron mobility of devices and reduce film cracking or peeling from the substrate. Thin film stress can be affected by many complex factors like microstructures and deposition parameters. Several complex ex-situ measurements and analyses can be ignored through this study of machine learning exercises.

**Figure 6:**
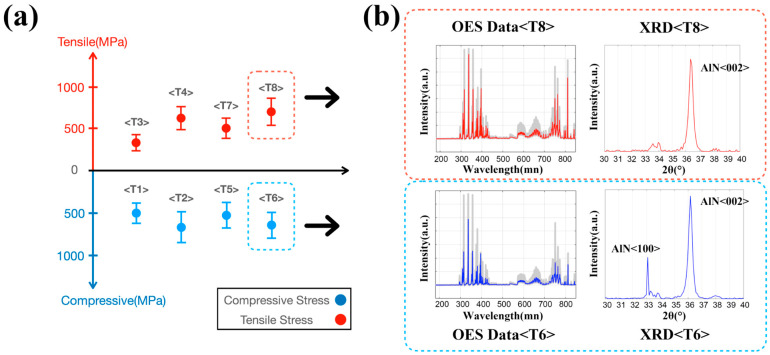
(**a**) Scatter plot with error bars calculated from XRD result reflecting eight groups (Table 1) of the experiment for MLP model training; (**b**) OES data plot (colored line: mean value of data, shadowed space: complete data) and XRD plot from 30 to 40 degree in 2θ diffraction angle of specific groups (T6 and T8).

In Figure 6a, compressive and tensile stress are obtained after experiments, and we can roughly judge that the residual stress has a noticeable difference with the change of N_2_ flow rate and power. Apparently, even with the increase of N_2_ flow rate and process power (see Table 1), the compressive stress changes to tensile stress (see Figure 6a). With the increase of the N_2_/Ar flow ratio and sputtering power, the thickness of AlN increases, indicating that the transition really exists from compressive stress to tensile [30]. Increasing the sputtering power energetically enhances the deposition rate, with the increased kinetic energy transferring to the sputtered atoms. With the sputtering power induced energy transfer, ionized radical mobility, and surface diffusion length also increase, leading to the increased possibility of atomic diffusion [31]. Furthermore, when Ar remains constant, with the increase of N_2_/Ar flow ratio, the proportion of N^+^ or N_2_^+^ increases with the increase of deposition rate [32]. Therefore, the above power and flow rate changes will be highly reflected in OES data through plasma composition and energy changes. In combination with the film measurements by XRD, SEM, and TEM, we can attempt to establish a connection between the OES data and the film results. Thus, machine learning becomes a great way to process complex OES data.

Figure 6b shows the OES data and XRD of two representative groups. The plot in the red dotted line represents group T6 with tensile stress, and the plots in the blue dotted line patterns represent group T8 with compressive stress. The overall emission intensity T8 is relatively strong from the OES data plots, but it is still challenging to find many differences. In group T8, only AlN (002) orientations that require energy accumulation appear at the 2θ diffraction angle between 30° and 40° due to the higher deposition power from the XRD plot. In contrast, in the T6 group, there is an apparent peak in the orientation of AlN (100).

### 3.2. Microstructure of Thin Film

The influence of process parameters on the film stress was mentioned above. However, the microstructure is more intuitive because we can discern the classification of stress and the correlation with the microstructure.

**Figure 7:**
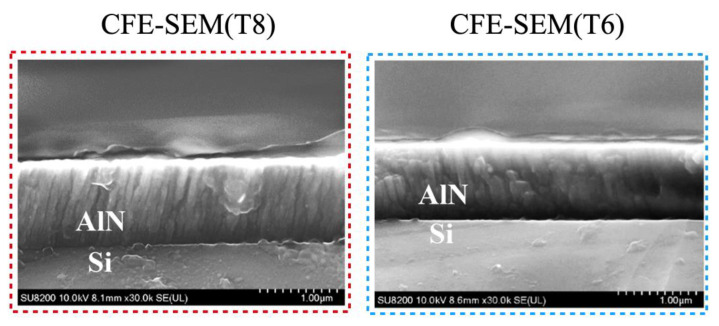
Cross-section CFE-SEM images and of a tensile group T8 and a compressive group T6.

In Figure 7, cross-section CFE-SEM images of group T8 (tensile) and T6 (compressive) are shown. The film thickness of T8 is thicker than that of T6 due to its higher deposition power, and the columnar structure in the CFE-SEM image can be easily observed. Differently, in Group T6, compared with T8, the columnar structure of AlN in CFE-SEM is not apparent [9]. This phenomenon can be explained by adatom mobility. At lower power, the radicals have less kinetic energy, resulting in lower adatom surface mobility. In general, higher adsorbed atom mobility promotes the growth of C-axis oriented AlN thin films because the higher the mobility, the more likely it is that low-energy binding sites leading to crystal growth will be found [10].

**Figure 8:**
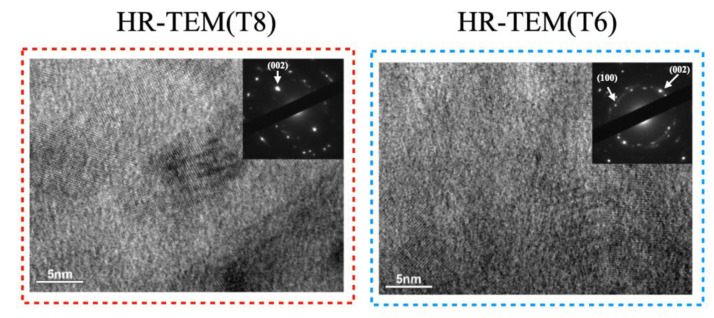
Bright field HR-TEM images with corresponding electron diffraction (SAED) pattern of a tensile group T8 and a compressive group T6.

In Figure 8, the corresponding electron diffraction (SAED) patterns of a tensile group T8 and a compressive group T6 are shown. In the HR-TEM images, the constitution of columnar grains was analyzed using bright field and SAED patterns. Along the c-axis, a textured growth can be observed. Like other wurtzite-type structures, planar defects like stacking and dislocation faults are observable in the bright field image. It can also be inferred from the SAED patterns that the arrangement of columnar grains is not perfect. Displacement of the (002) reflection can be found [12]. In the bright field image of group T6, compared with the T8, the texture seems disorderly. The SAED pattern is also a more apparent polycrystalline structure consistent with the result of complicated crystallization in XRD.

It is not difficult to conclude that there is a correlation between the complex microstructure and differing film stress. It can be seen from the SEM results that the growth of the crystal can lead to the change of stresses, and TEM results (Figure 8) show that our XRD measurement is effective. Apparently, this is a merit that, from this study, we are encouraged to bypass these complex ex-situ film analyses and resolve film stress issues through machine learning without taking too much time for analysis and validation.

### 3.3. Model Preparing and Performing

After completing the stress measurement, we package eight OES data groups and divide them into compressive stress and tensile stress for PCA (principal component analysis) and MLP (multilayer perceptron). We need to consider which PCs (principal components) are needed to feed to MLP and what kind of hidden layers structure we need to define our training model. To deal with the input neurons of PCA, we first calculate the contribution of these data in different PCs. The percentage of variance of these groups of data from PC1 to PC5 are 88.0%, 8.9%, 1.5%, 1.0%, and 0.4%, respectively. It can be found that the cumulative rate of the first three PCs can reach 98.4%. The first three PCs are enough to represent complete data, and in order to avoid irrelevant OES noise being used for classification, we only consider using PC1 to PC3 [22].

It has been shown that MLP is a universal approximator. As long as there are enough hidden nodes, MLP can approximate any needed function. In practice, it is also hard to know how many nodes are needed and to be used in hidden layers of a given problem. We do not know how to learn or set its weight effectively. However, the single hidden layer solution is very inefficient [29]. Through many tests, we select [6-3] (which means six nodes in the first hidden layer, three nodes in the second hidden layer) and [9-6-3] for our consideration of hidden layers as nodes in the first, second, and third hidden layer. In the process of training, we applied the Levenberg–Marquardt algorithm (LMA) as our training model. Although this method requires more computing power, it is the fastest one for training, and it is worthy for thousands of training times [33].

As shown in Table 2, after identifying several preferred neurons (PCs or complete spectrum) as well as input and hidden layer structures, we want to know how the formed MLPs perform. After processing these formed MLPs, we obtain some indicators for further consideration. At the same time, we also confirm whether we should feed the data into MLP after PCA processing. We used the complete spectrum (1900 raw spectrum point) that PCA did not process as input neurons. These models are trained by MATLAB 2021a in this study using build-in ANNs functions. The percentage of exemplars for training is 70%, 15% is for training cross-validation, and 15% is for testing.

We use accuracy (ACC), root mean squared error (RMSE), and epoch to judge to find the most suitable training model for our experiment [33]. *ACC* is the probability that the training model can correctly classify the input contents, and the equation is as follows:

(2) $$\begin{array}{c} \;\;Acurracy\left(ACC\right)=\frac{TP+TN}{P+N}\; \end{array}$$

where true positive (*TP*) and true negative (*TN*) represent cases where the prediction results are consistent with the actual situation, and the value of *P* + *N* represents the whole training samples. RMSE is a convenient method to measure “average error” and is calculated by the difference between the true condition and the predicted condition, and the sum is averaged and then is rooted. Equation of *RMSE* is given as follows:

(3) $$\begin{array}{c} \;\;\;RMSE=\sqrt{\frac{\sum_{i=1}^{n}{\left(y_{i}-{\hat{y}}_{i}\right)}^{2}}{n}}\; \end{array}$$

where *y* is an estimated parameter and *ŷ* is an estimator, and the value can be 0 or 1 based on whether it is compressive or tensile. The smaller the *RMSE* value is, the better the accuracy of the prediction model to describe experimental data [34].

When complete data passes through the MLP once and returns, this is called an epoch. In these pieces of training, the maximum epochs are set to 1000, and the “epoch #” in the table indicates how many times you have trained to achieve the best performance. However, there is no conclusion regarding how many epochs are suitable, but as the updating time neural network increases, the curve to classify changes from underfitting to overfitting.

Although the import of complete spectrum (1900 neurons without PCA) has a fragile advantage, we also found that it takes about 250–300 times more in actual model training time and about 25 times more in model building time (building time includes the time of data loading data processing, model training, and model output). The above time results were tested on the Apple M1 SoC (silicon on chips) platform with 16 GB memory, and the training time can be saved on the better performing device. However, we believe that the general performance device is more in line with the actual application situation of the semiconductor or related industries as well as cost considerations.

### 3.4. Stress Perdition and Verification Result

**Figure 9:**
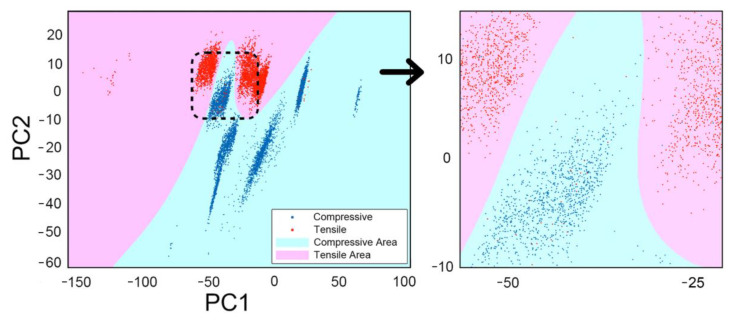
Performed model using a [9-6-3] MLP with PC1 and PC2 input, and successfully classified the stress as compressive or tensile.

After various considerations, we finally chose the [9-6-3] MLP with PC1 and PC2 input for our model. Fortunately, the input of PC1 and PC2 can be well rendered by using a plot. As shown in Figure 9, the junction of the red area and the blue area is the classification result trained by machine learning. Because of the dense data point, we also plot a local enlarged image on the right. The blue dots represent the neurons with compressive input and the red dots represent tensile input. If dots appear in the area of the same color, this model is correct for the input neuron classification. Otherwise, the model is not able to distinguish correctly.

**Figure 10:**
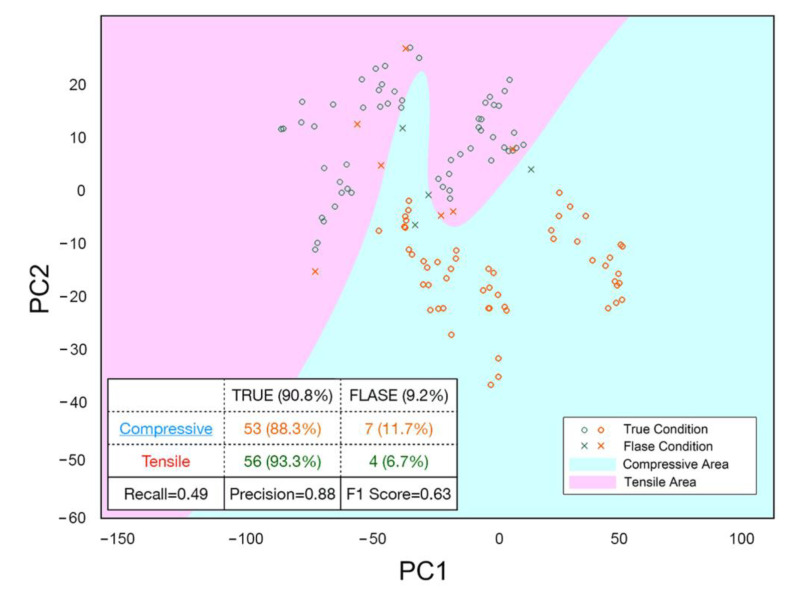
Cross-validation test result with a confusion matrix.

However, we desired to know whether this model can be applied under other experimental conditions. We have two variables (nitrogen flow rate and sputtering power) in the training experiment, each with four values. In designing the verification experiment, we randomly combined the two variables. We collected the spectrum in the following experiments with different N_2_ flow rates and power. After the film measurement, we randomly selected spectrums from verification experiment V1-V16, as shown in Table 3, to evaluate the model’s actual performance. Among them, the prediction of 60 random experiments was compressive, and the prediction of the other 60 random experiments was tensile. We use PCA to process these random cross-validation spectrums with the previous OES data, then plot them on the previously trained model, as shown in Figure 10. Green points represent tensile perditions and orange point represents compressive predictions. If the prediction is correct, the points are plotted as circles. The points are plotted as crosses on the contrary.

The table in Figure 10 presents a confusion matrix on the cross-validation test performance. A confusion matrix is typically used in supervised learning with visualization. It is so named because it is easy to see whether the machine has confused two different classes through this matrix. We can receive the values of recall, precision, and F1 score to score this model through calculation. The recall is the proportion of positive samples that can be predicted among all positive samples, and the precision is the proportion of positive samples among all positive samples. F1 score is the harmonic average of the two, which can be regarded as a good general index to see the performance of this model [21]. We are pretty satisfied with the performance of this model. At present, there are only 10,000 sets of training data. We believe that thin film stress classification will become more effective if more data are used during training of the model.

## 4. Conclusions

In this study, we have carried out eight groups of experiments to study the effect of stress obtained using XRD measurement and correlating with TEM microstructures on AlN thin films processed by a pulsed DC reactive sputtering with an in-situ OES. With different experimental conditions, we observed the transformation of stress from compressive to tensile. Stress is a very complicated physical characteristic affected by many factors, such as deposition parameters (i.e., power and gas flow) and film microstructures. However, we have proven that machine learning combining PCA with ANNs can solve the semiconductor process problems more efficiently. Combined with PCA, the prediction time is highly preserved with a shallow loss of accuracy. In recent years, machine learning has become a significant trend. Apparently, this is a merit that we have bypassed with these complex ex-situ measurements and analyses on thin films to create a method for in-situ stress prediction and classification with in-situ OES data. We also expect that these methods can be further extended and could be beneficial to the semiconductor and related industries in the future.

## Figures and Tables

**Table 1:** Experimental parameters of experiment T1–T8.

	Ar Flow Rate (sccm)	N2 Flow Rate (sccm)	Sputtering Power (W)
T1	15	15	800
T2	15	30	800
T3	15	45	800
T4	15	60	800
T5	15	45	400
T6	15	45	600
T7	15	45	800
T8	15	45	1000

**Table 2:** Modify the number of input neuron(s) and the composition of the hidden layer(s) for training and discuss the performance of the models.

Input Neuron(s)	Hidden Layer(s)	ACC (%)	RMSE	Epoch #	Building Time
1 (PC1)	2 [6-3]	94.83	0.202	63	29 s
1 (PC1)	3 [9-6-3]	94.88	0.187	32	31 s
2 (PC1&2)	2 [6-3]	98.79	0.172	34	30 s
2 (PC1&2)	3 [9-6-3]	98.97	0.0577	25	33 s
3 (PC1-3)	2 [6-3]	98.96	0.0996	47	33 s
3 (PC1-3)	3 [9-6-3]	98.97	0.0947	40	34 s
1900 (without PCA)	2 [6-3]	99.10	0.00269	12	12 m 21 s
1900 (without PCA)	3 [9-6-3]	99.10	0.00288	13	13 m 30 s

**Table 3:** Experimental parameters of cross-verification experiment V1–V16.

	400 W	600 W	800 W	1000 W
15 sccm	V1	V2	V3	V4
30 sccm	V5	V6	V7	V8
45 sccm	V9	V10	V11	V12
60 sccm	V13	V14	V15	V16

## Data Availability

The data presented in this study are available on request from the corresponding author. The data are not publicly available due to the data sets are too large in this study.
